# Filament Dynamics during Simulated Ventricular Fibrillation in a High-Resolution Rabbit Heart

**DOI:** 10.1155/2015/720575

**Published:** 2015-10-26

**Authors:** Pras Pathmanathan, Richard A. Gray

**Affiliations:** U.S. Food and Drug Administration, 10903 New Hampshire Avenue (WO 62), Silver Spring, MD 20993, USA

## Abstract

The mechanisms underlying ventricular fibrillation (VF) are not well understood. The electrical activity on the heart surface during VF has been recorded extensively in the experimental setting and in some cases clinically; however, corresponding *transmural* activation patterns are prohibitively difficult to measure. In this paper, we use a high-resolution biventricular heart model to study three-dimensional electrical activity during fibrillation, focusing on the driving sources of VF: “filaments,” the organising centres of unstable reentrant scroll waves. We show, for the first time, specific 3D filament *dynamics* during simulated VF in a whole heart geometry that includes fine-scale anatomical structures. Our results suggest that transmural activity is much more complex than what would be expected from surface observations alone. We present examples of complex intramural activity, including filament breakup and reattachment, anchoring to the thin right ventricular apex; rapid transitions among various filament shapes; and filament lengths much greater than wall thickness. We also present evidence for anatomy playing a major role in VF development and coronary vessels and trabeculae influencing filament dynamics. Overall, our results indicate that intramural activity during simulated VF is extraordinarily complex and suggest that further investigation of 3D filaments is necessary to fully comprehend recorded surface patterns.

## 1. Introduction and Background

Atrial and ventricular fibrillation are highly complex processes, whose mechanisms are still not well understood. Since the structures of the atria and ventricles are very different—for example, the atria contain many orifices capable of supporting anatomical reentry—the effects of geometrical factors on fibrillation are expected to be quite different [1]. In this paper, we focus on ventricular fibrillation (VF) exclusively. There are immense technical challenges in experimentally measuring the electrical activity during VF, due to its complexity and spatially distributed nature. Regarding surface activity, experimental techniques such as optical mapping [2], epicardial socks/plaques [3], and endocardial balloon/basket electrode arrays [4] are used to record electrical activity from the heart surface. In contrast, while some investigators have measured transmural activation patterns during VF [5, 6], much remains unknown regarding intramural activity and its role in maintaining VF. Understanding the intramural wave dynamics is vital to the development and refinement of preventative and therapeutic measures for VF, an event which causes death within minutes without intervention, and a significant contributor to sudden cardiac death being the leading cause of fatality in the western world.

Computational modelling enables visualisation and analysis of electrical activity throughout the full 3D heart at nearly cellular resolution and also the specification and control of factors (e.g., geometry or cell dynamics) that would be impossible in the experimental and clinical settings. As such, computational modelling of cardiac electrophysiological activity is a thriving field, which has its roots in Nobel Prize winning work, namely, the development of the Hodgkin-Huxley model governing neuronal electrical activity [7]. Much research has been devoted to developing a complete model of the isolated cardiomyocyte, and there are currently over a hundred of these “cell models” [8]. They can be used to reproduce action potentials and other cellular and subcellular phenomena or used in whole-organ simulations by being coupled to equations governing spatial propagation of electrical waves and solved on a suitable geometrical representation of the heart. Unfortunately, these cell models have not been rigorously validated and in some cases similar models produce dissimilar predictions [9–12]. Developing a credible organ-level model of ventricular fibrillation, in particular for the diseased human heart, is one of the greatest challenges in cardiac modelling. This will include a comprehensive understanding of the underlying mechanisms of VF, as well as identifying contributing factors, and their relative roles. Due to the complexity and variability of the human disease state and the difficulty in obtaining data from patients, many believe that integrating animal experiments with computer simulations and theoretical analysis is necessary to develop the required comprehensive understanding. Here, we develop and analyse a model of rabbit VF. Fibrillation in the human heart is thought to be more similar to that in the rabbit than other large mammals ([13, 14], discussed further below). This choice enables us to study the impact of fine-scale structure, which would not be possible with current computational meshes of the human heart.

Ventricular fibrillation is generally believed to be the result of multiple unstable reentrant waves moving through the entire heart. Unlike anatomical reentry, in which the electrical wave continuously rotates around a fixed obstacle (e.g., the mitral valve) in a stable manner (resulting in rapid periodic activity in which the period is related to the perimeter of the obstacle), the activity during VF is aperiodic and results from multiple unstable functional reentrant circuits within the heart muscle. The similarity of reentry in thin 2D slices of cardiac tissue [15] to “spiral waves,” which have been studied analytically by mathematicians [16–20], stimulated much cross-disciplinary investigation and led to new methods for analysing the complex spatiotemporal patterns observed during VF and new insights into VF mechanisms. For example, Gray et al. [21] introduced the concept of a “state-space encoded phase variable,” *θ*, which is an angular representation of the action potential. This representation provided a means to identify the number and location of unstable reentrant waves on the heart surface during VF, via the identification of* phase singularities* (PSs), the point in 2D space around which a spiral wave rotates, computed from the spatial maps of *θ*. It also provided a formal link to mathematical theory, and hence the general and qualitative notion that VF results from “random meandering wavelets” has been largely replaced in the scientific community with the idea that “multiple unstable spiral waves” are the underlying mechanism of VF.

In 3D, reentrant waves are actually “scroll waves,” which rotate around a 1D curve in 3D space, called a* filament*. Filaments follow some topological constraints [22, 23]; for example, a filament must either end on a boundary or form a closed curve. As opposed to surface phase singularities, the behaviour (and number) of filaments in the whole heart during VF is largely unknown, and therefore simulations have the potential to help elucidate filament dynamics.

Simulations of scroll waves in 3D tissue slabs have increased our understanding of filament behaviour [24–28]. Certain simple filament shapes have been observed with various cellular models (many noncardiac). These include* linear* (or near-linear) transmural filaments, associated with one epi- and one endocardial phase singularity;* U-shaped* filaments, in which the two PSs at the filament ends reside on the same heart surface (either epi- or endo-); and* O-shaped* filaments, in which the filament is comprised of a closed curve (i.e., a ring) residing completely within the heart (no surface PSs). One very important, uniquely 3D, characteristic of reentry is a feature called* filament tension* [29]. According to theory [30], tension determines the fundamental behaviour of whether a filament grows or shrinks. It was originally believed that because of the “high excitability” of cardiac tissue, filament tension in healthy tissue should be positive (corresponding to shrinking of filaments). For ischemia, in which excitability is drastically decreased, negative filament tension could be explained. Under positive tension, O-shaped filaments will shrink and self-annihilate, and curved filaments will evolve to minimise filament curvature. More recent evidence [31], however, suggests that this initial view regarding filament tension may have been too simplistic. Another uniquely 3D mechanism of scroll wave instability involves the high wavefront curvature resulting from the twisting of fibre rotation across the ventricular wall. Fenton and Karma [25] described how phase shifts of spiral wave rotation across the wall can lead to significant transmural gradients of transmembrane potential resulting in* twistons* that propagate along the filament and sometimes break off forming new filaments.

Filament analyses have also been performed using whole heart geometries, for example, [14, 28, 32–36]. However, while detailed quantification of* global metrics* has been performed at the whole heart level, as far as we are aware, very little information is available on filament* dynamics* in simulations using physiological cell models and realistic heart geometries and none with detailed heart structure. For example, ten Tusscher et al. [14, 32] quantified the number of filaments in various computational models of mammalian VF. Their work supports the theory that VF in the human heart is more closely related to VF in the rabbit heart, in terms of spatial organisation, than other large mammals (e.g., dog/pig) [13]. Clayton [28] presents a detailed analysis on how numerous metrics (including number of filaments, lifetimes, number of births, deaths, and divisions) are affected by membrane kinetics and geometry. Arevalo et al. [35] and Trayanova et al. [34] present postshock filament distributions, and some filament dynamics are described, although not in detail. All of the above studies use representations of the heart that* do not include fine-scale structure*. Bishop et al. [37] have developed a high-resolution, anatomically detailed computational model of the rabbit heart that includes structures such as large intramural vessels, papillary muscles, and trabeculae. Bishop and Plank [36] then studied the role of this structure in rabbit ventricular tachycardia (VT) and fibrillation, with comprehensive analyses using metrics such as number of surface PSs, number of filaments, and spatial distribution of cumulative filament count. They conclude that structure has little effect on rabbit arrhythmia maintenance, although this is in contradiction to some experimental findings with larger mammals [38–41].

As far as we are aware, there has never been a detailed presentation of filament dynamics in whole heart simulations with highly anatomically detailed computation meshes. In this paper, we present a model of VF composed of a novel cellular model and the Bishop et al. anatomically detailed mesh of the rabbit heart [37] and fill this gap in the research literature by describing various filament dynamics during simulated VF. Where appropriate, we show the corresponding surface patterns, and in particular we will see that the activity in the interior of the heart may be far more complex than what would be assumed given the surface patterns alone.

## 2. Simulating Ventricular Fibrillation

To simulate VF we used a recently developed cell model of electrophysiological activity of the cardiomyocyte [42] and coupled this to established equations governing propagation of electrical waves through cardiac tissue and computed electrophysiological activity on a highly anatomically detailed computational mesh of the rabbit ventricles, as described below.

A “natural” choice of cell model would be a recent physiological rabbit cell model, for example, the Mahajan et al. [43] cell model; however, Bishop and Plank [36] had to alter ion channel kinetics and tissue conductivities in order to simulate VF with this model. Similarly, both Bishop and Plank [36] and Pathmanathan and Gray [44] had to decrease tissue conductivities (effectively reducing the wavelength) with this cell model to simulate VT. As far as we are aware, there are no published studies of simulated VF in the rabbit heart using an unaltered ionic model and realistic tissue conductivity values. In contrast to previous studies in which VF was simulated using phenomenological [28] cell models, cell models from other species [45], or altered cell models/tissue parameters [36], we made use of a realistic rabbit fast sodium current (*I*_Na_) model and realistic tissue parameters and obtained VF by controlling repolarisation dynamics. Specifically, we used a three-variable (voltage *V*, activation variable *m*, inactivation variable *h*) Hodgkin-Huxley (HH) *I*_Na_ model which we have recently developed using multiscale data [42], for which the *I*_Na_ current matches that measured experimentally in the whole rabbit heart during propagation while pacing at 300 ms. The total ionic current is then taken to be the sum of this *I*_Na_ and a simple phenomenological repolarisation current [46], as follows:

(1) $$\begin{array}{c} I_{\text{ion}}=I_{\text{Na}}+g\left(V-V_{\text{rest}}\right)e^{-\beta (V-V_{\text{rest}})}, \end{array}$$

where *g* = 0.5 *μ*A/(cm^2^·mV), *V*_rest_ = −83 mV, and *β* = 0.035/mV. It is important to note that this hybrid HH-phenomenological model cannot be used to study all cellular phenomena. For example, since the repolarisation current contains no gating variables, it does not exhibit rate dependence of repolarisation and in particular cannot reproduce action potential duration (APD) restitution. However, its simplicity does provide a straightforward means to study the interactions of the wavefronts and wavetails, through control of the parameter *β* which determines APD without altering *I*_Na_ kinetics. VF was obtained through reduction of APD; this approach is similar in philosophy (but not implementation) to others who have adjusted both cell model dynamics and/or conductivities as described above and has the advantages of realistic* wavefront* dynamics. Like previous work, our simulated VF exhibits a shorter wavelength than that observed experimentally (see Section 3), but we believe this does not affect the overall inferences of this paper, as discussed in Section 5.

Spatiotemporal electrophysiological activity in the whole heart can be modelled using the bidomain equations [47]: two partial differential equations (PDEs) coupled to a choice of cell model, governing transmembrane and extracellular potentials. Alternatively, the monodomain equations [47], a simplification of the bidomain equations to a single PDE, may be used and typically provides a reasonable approximation in the absence of extracellular phenomena such as defibrillation shocks. For this paper we used the monodomain equations, coupled to the cell model (1):

(2) $$\begin{array}{c} \chi \left(C_{m}\frac{\partial V}{\partial t}+I_{\text{ion}}\left(u,V\right)\right)-\nabla \cdot \left(\sigma \nabla V\right)=0, \end{array}$$

where **u** = (*m*, *h*) are the state variables of the cell model, *χ* = 1400  cm^−1^ is the surface-area-to-volume ratio, and *𝒞*_*m*_ = 1.0 *μ*F·cm^−2^ is the capacitance per unit area. The conductivity tensor *σ* was taken to be transversely isotropic, with values *χ𝒞*_*m*_ *D*_*L*_ in the fibre direction and *χ𝒞*_*m*_ *D*_*T*_ in the cross-fibre directions, where *D*_*L*_ = 0.001 cm^2^·ms^−1^ and *D*_*T*_ = *D*_*L*_/9, chosen to match fibre and cross-fibre conduction velocities on the epicardial surface of the rabbit heart [48]. The equations were solved using the finite element method, using the software package “Chaste” (Cancer, Heart and Soft Tissue Environment) [49]. The cardiac electrophysiological component in Chaste is a powerful, highly optimised suite of libraries for computing electrophysiological activity under various formulations. The accuracy and reliability of Chaste have been very heavily tested, and as part of a recent work on* verification* (defined as confirmation that a computational model (software) correctly solves an underlying mathematical model), we have developed monodomain and bidomain problems with exact solutions in 1D, 2D, and 3D and confirmed that Chaste correctly solves these problems, with convergence rates predicted by theory [44].

**Figure 1:**
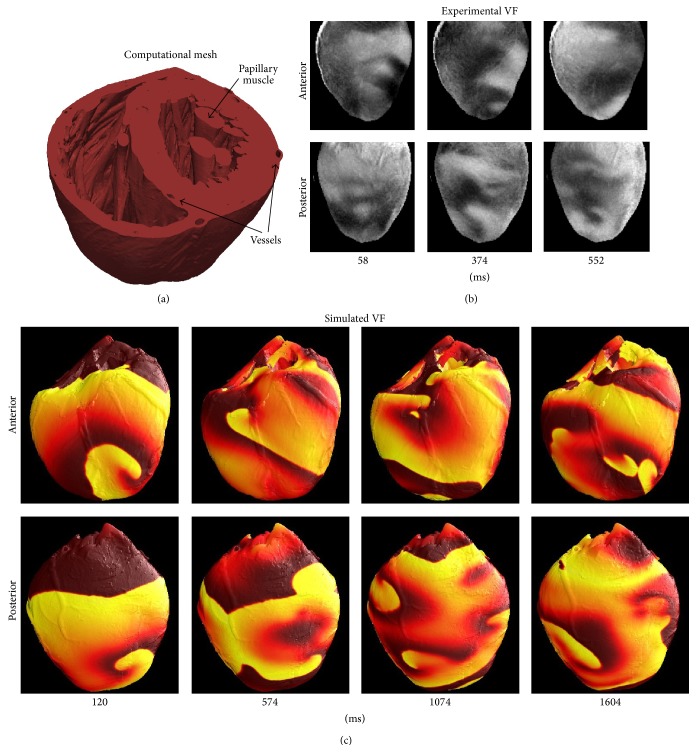
(a) High-resolution (21 million tetrahedral elements), anatomically detailed computational mesh. (b) Optical mapping recordings showing snapshots of VF in isolated rabbit heart. (c) Surface snapshots of simulated VF, with colour representing transmembrane voltage.

The monodomain equations and cell model (1) were solved on the high-resolution, highly anatomically detailed, computational mesh of the rabbit ventricles [37], which is comprised of 4.1 million nodes and 24 million elements, and have an average edge length of 125 micrometres. As stated above, this mesh includes structure such as large transmural vessels, papillary muscles, and trabeculae and is illustrated in Figure 1.

To obtain VF, an initial stimulus was applied near the apex which generated a wave that propagated upward toward the base, followed by an S2 stimulus applied in a large ellipsoidal region on the lateral posterior wall of the left ventricle, timed to overlay the apex-to-base repolarisation wave. (Stimuli were applied by clamping transmembrane voltage rather than addition of a stimulus current; this is necessary to prevent unphysiologically long action potentials given the simple phenomenological repolarisation current used.) This induced two counter-rotating waves that evolved into ventricular fibrillation. Insulating boundary conditions were enforced. The monodomain partial differential equations (PDEs) were discretised using a PDE timestep of 0.1 ms. In each 0.1 ms time increment, the voltage is constant, and, due to the simplicity of the cell model—as mentioned above the only other variables in the cell model are the *I*_Na_ gating variables *m* and *h*—the differential equations for *m* and *h* can be solved analytically in each time increment. Therefore, no ordinary differential equation (ODE) solver is required. Due to the high resolution of the mesh and the long duration of the simulation (2 seconds), simulations are computationally demanding. They were performed on an FDA cluster comprised of more than 3,000 CPU-cores and a 40 Gbps InfiniBand fabric for internode communications and required approximately 40 minutes computation time per second of fibrillatory activity using 128 processes. It should be noted that simulations were limited not by computational cost but by the large datasets (e.g., 32 GB) that required postprocessing.

Filaments were identified at each time instant via a two-stage procedure. First, phase was computed at each node of the mesh as done previously [21], using the state-space encoded phase variable *θ* defined as

(3) $$\begin{array}{c} \theta \left(x,t\right)=\arctan\left(\frac{V\left(x,t+\tau /2\right)-V_{\text{ref}}}{V\left(x,t-\tau /2\right)-V_{\text{ref}}}\right) \end{array}$$

(using the arctan function that takes quadrant into account, i.e. “atan2”), where *V*_ref_ = −30 mV and *τ* = 4 ms. Second, every face of every element in the computational mesh (the mesh is comprised of tetrahedral elements and each element therefore has four triangular faces) was analysed to determine if a phase singularity was present within the face. This was done by summing the differences Δ*θ* in phase across each of the three sides of the triangle, with all Δ*θ* converted to a value in the range [−*π*, *π*) before contributing the sum. The sum is equal to zero if no PS is present; otherwise, it is equal to ±2*π*. Each tetrahedral* element* was found to have exactly zero or two triangular faces with phase singularities, as expected. When two faces with phase singularities were detected, the centroids of the two faces were computed, and the filament segment was defined to be the line connecting the two centroids.

## 3. Surface Electrical Activity

Snapshots of the electrical patterns during VF on the anterior and posterior surfaces of the heart are shown in Figure 1, both for simulations and for experiments in the isolated rabbit heart, and the latter obtained using a dual-camera fluorescent imaging system [21]. In both experiments and simulations, the epicardial surface patterns were characterised as never-repeating complex patterns with short-lived reentrant waves (evident as PSs) and target patterns. Reentrant waves that lasted for more than one rotation (i.e., rotors) were evident but relatively infrequent. Due to the decreased repolarisation dynamics in the model as discussed in Section 2, the frequency of activity in the simulation (about 28 Hz) is greater than that recorded in optical mapping experiments (about 10 Hz [21]). However, it is important to note that drugs used in optical mapping experiments to eliminate the contractions of the heart, which would interfere with high spatial-resolution imaging [2, 21], affect important electrophysiological properties such as APD and the dynamics of arrhythmias [50]. Samie et al. [51] have measured the frequency during VF in the isolated rabbit heart without anticontractile drugs to be 13–16 Hz, which is somewhat closer to the simulated frequency, although of course, it is still quite different. The use of decreased cycle lengths and wavelengths in simulations of VF will be discussed further in Section 5, together with evidence that the cycle length does not affect the inferences drawn in this paper.

## 4. Subsurface Electrical Activity

As far as we are aware, the specific behaviour of filaments during VF in biventricular simulations utilising anatomically detailed computational meshes has never been published. In this section, we present several examples of such filament dynamics. In general, filament dynamics were quite complex; multiple unstable 3D reentrant waves continuously broke up and self-terminated. We have chosen several examples which we believe are representative of the simulations.

**Figure 2:**
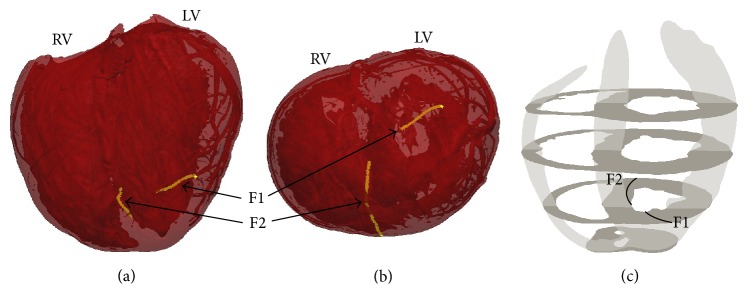
Two filaments following the S2 stimulus in which two counter-rotating reentrant waves were present, before VF has developed. (a) Anterior, inclined toward apex, view of the computational mesh (translucent so that fine-scale structure such as coronary vessels can be seen), with two filaments shown in gold. (b) Apical view (apex-to-base view). (c) Schematic of the biventricular geometry and approximate location of the filaments.

We begin with a simple example in Figure 2. Here, the filaments shortly after the S2 stimulus are shown, when two large epicardial spiral waves are present (cf. Figure 1, simulation at 120 ms) but before VF has developed. (The mesh is shown as translucent, which allows some of the fine-scale structure to be observed, such as coronary vessels in the lateral left ventricular wall). In this example, only two filaments are present.

**Figure 3:**
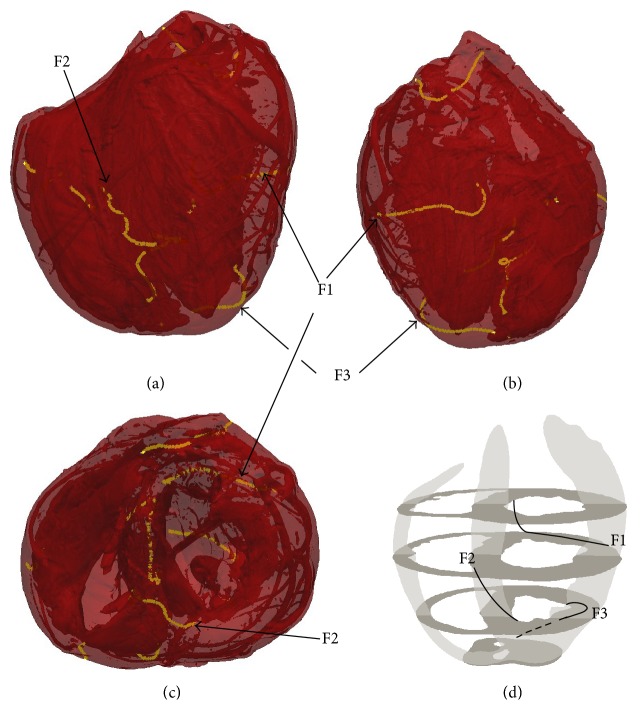
Various views ((a) anterior; (b) posterior; (c) base-to-apex; (d) schematic) of the heart at a moment in time (686 ms) after VF has developed when multiple long filaments are present. Three filaments are highlighted.

Figure 3 is a snapshot of filaments after VF has developed, when spatial organisation is more complex. We show this example to illustrate that the length of filaments can be quite long—much longer than the wall thicknesses of the RV, LV, and septum. One end of the filament labelled F1 is on the epicardial LV free wall and thus will be seen as a PS on the LV free wall surface, while its other end is on the LV endocardium but near the septum; hence, the filament spans the entire posterior LV transmurally. Filament F2 spans the septum with one end on the RV side (about midway from base to apex) and the other end on the LV side closer to the apex. Obviously, this filament does not exhibit any surface PSs; as a reentrant wave it contributes to sustaining VF, but it is not observable as reentry on the surface. Filament F3 exhibits one surface PS, with one end on the endocardium of the LV free wall and the other on the LV epicardium near the apex.

**Figure 4:**
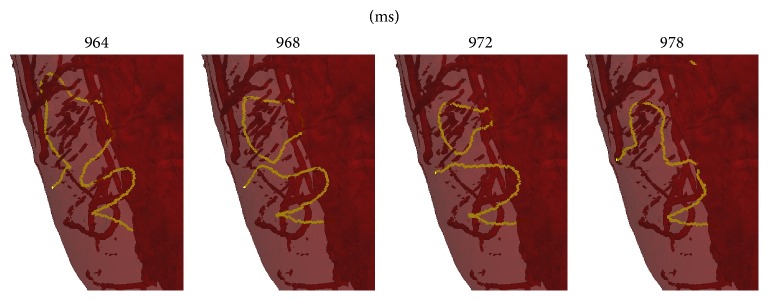
Close-up of intramural filament dynamics. Initially (*t* = 964 ms), a single long convoluted epi-to-endo filament is present. It then breaks up into a shorter epi-to-endo filament and a ring (*t* = 968 ms; note that the ring is partially obscured). The ring begins to shrink (*t* = 972 ms) but then reconnects to the epi-to-endo filament (*t* = 978 ms).

Filaments during simulated VF were not static but continuously evolved by changing shape, interacting, and self-terminating.  Figure 4 illustrates an example of such transient behaviour. Initially, an extremely long single filament winds from the LV epi- to endocardium. As this filament evolves, it “intersects” itself giving rise to a ring filament and a shorter epi-to-endo filament. The ring begins to shrink but then reattaches to the transmural filament, once again resulting in a single long epi-to-endo filament.

**Figure 5:**
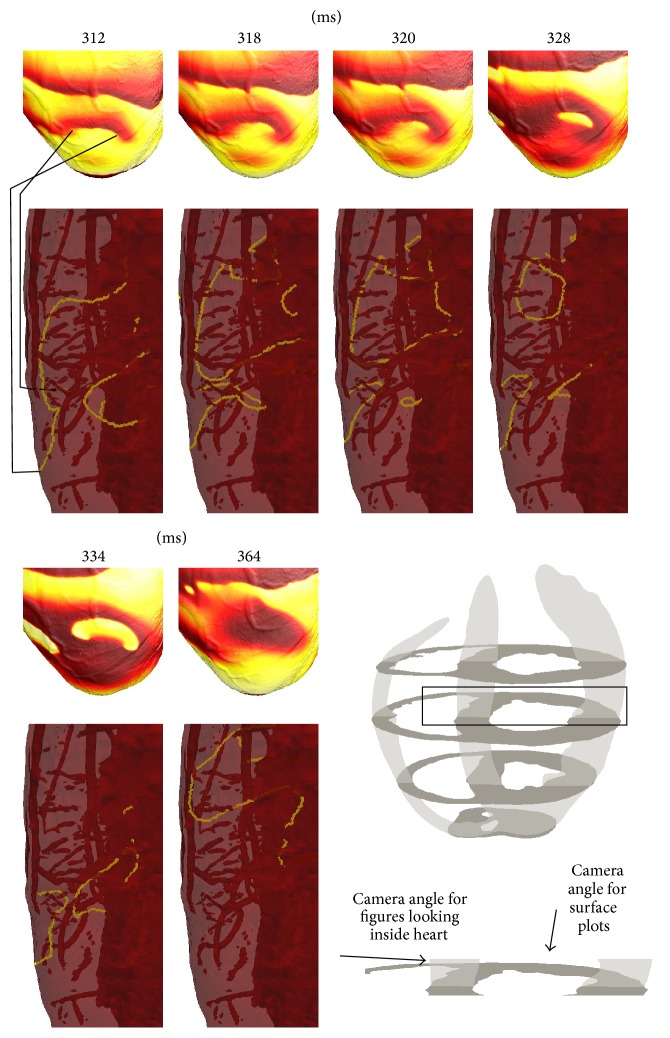
Surface manifestation of filament dynamics.* Upper*: surface patterns of transmembrane voltage.* Lower*: corresponding side-on view of filaments below the surface.* Bottom right*: schematic showing location of interest in the heart, with arrows showing the angle of view for the surface and filament panels. In the first pair of figures, lines connect the phase singularities in the surface plots with the corresponding filament ends in the 3D view (note that the filament corresponding to the left phase singularity is mostly obscured). The filaments exhibit complex behaviour that is not evident from the surface patterns—see text for discussion.

To illustrate how complex filament shapes manifest on the epicardial surface, we now present examples which show both surface activity and the corresponding underlying filament dynamics.  Figure 5 illustrates that complex subepicardial behaviour is not always apparent from the surface patterns. For the first five surface snapshots, two phase singularities are observed which move across the surface and might be expected to correspond to one U-shaped epi-to-epi filament or two roughly linear epi-to-endo filaments. At the time of the first snapshot, the PS on the right is due to one epi-to-endo filament, while the PS on the left is due to a spatially distant second filament (mostly obscured in the image). There is also an endo-to-endo filament underneath the surface. Six milliseconds later, the filament configuration is completely different, with two closely spaced transmural epi-to-endo filaments (corresponding to the two PSs) and one large U-shaped endo-to-endo filament. Despite its close proximity to the surface, there is no evidence of reentry on the surface corresponding to the endo-to-endo filament (perhaps due the wavefront orientation along the filament). The two epi-to-endo filaments reform into one epi-to-epi and one endo-to-endo filament (*t* = 320 ms); then, the large endo-to-endo filament becomes a scroll ring (*t* = 328 ms) which shrinks and self-annihilates (*t* = 334 ms). After further transient activity (not shown), no filaments intersect with the epicardial surface and no epicardial PSs are observed (*t* = 364 ms).

**Figure 6:**
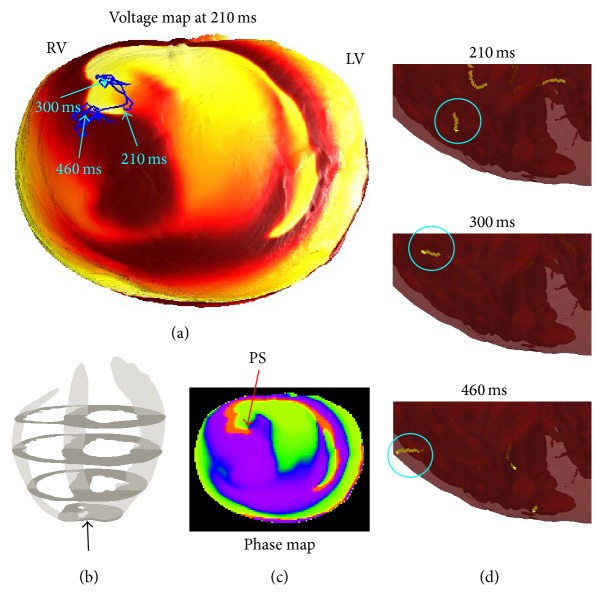
A small filament anchored to the right ventricular apex. (a) Surface potential at *t* = 210 ms, with an apex-to-base view. The surface phase singularity persists in this neighbourhood for 270 ms; the blue line plots its trajectory. (c) The spatial distribution of phase *θ* at *t* = 210 ms (values ranging from −*π* to *π*). The PS, identified by the arrow, is clearly visible. (d) Snapshots of the small stable transmural filament that corresponds to this phase singularity (movie also provided in supplementary material).

**Figure 7:**
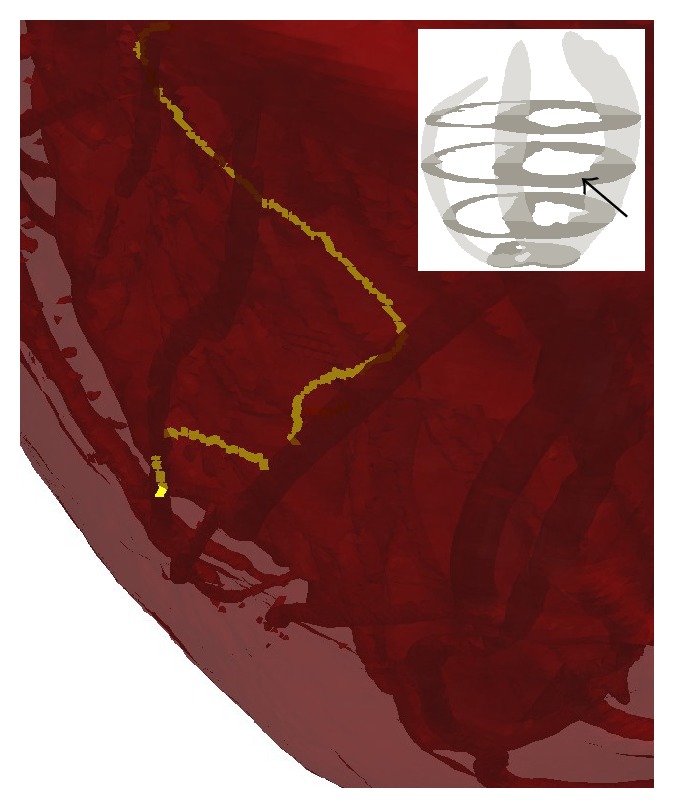
A snapshot showing a filament interacting with two coronary vessels, taken from a movie provided in the supplementary material. See text for discussion.

The simulations did not exhibit any sustained anchoring to any fine-scale structures; however, some episodes in which structure seemed to influence filament dynamics were observed.  Figure 6 displays the time evolution of a small transmural filament spanning the right ventricular wall near the apex. This filament is particularly stable, lasting about 280 ms; we consider this as anchoring to the right ventricular apex. A movie is also provided in the supplementary material (see Supplementary Material available online at http://dx.doi.org/10.1155/2015/720575). In addition, we observed various possible incidences of vessels affecting filament dynamics, as opposed to filaments travelling straight through vessels without being affected by the obstacle. In a movie provided in the supplementary material (see Figure 7 for a snapshot from this movie), we show a filament that travels through the myocardium and appears to linger on coronary vessels. While this evidence is anecdotal and has not been rigorously analysed, it may be indicative of structure affecting VF in a more subtle way than the very long timescale anchoring to arteries that has been observed in the experimental setting with larger mammals (see e.g., [38]).

**Figure 8:**
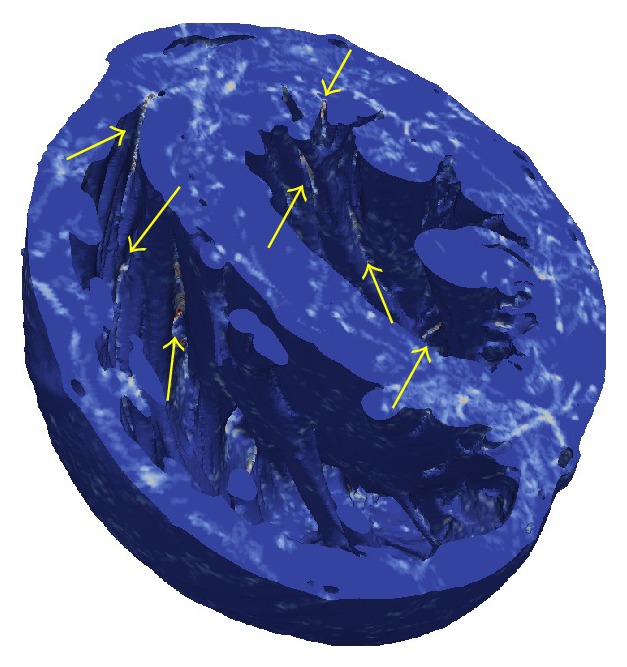
Filament density—the number of times when any element in the mesh contained a filament, over the course of the 2-second simulation. Colour scale ranges from blue (element never contained filaments) through white to red (element contained filaments for 50–75 ms). Multiple regions between endocardial trabeculae with higher filament density are indicated by arrows.

To search for evidence of anchoring or regions of increased filament clustering, we computed the filament density over the entire 2-second episode (computed by summing the number of times when any element in the mesh contained a filament). This is plotted in Figure 8. While no clear evidence of anchoring or clustering around vessels was found, the figure does show numerous regions of high filament density between endocardial trabeculae as indicated by the arrows. (Similar clustering, though perhaps slightly less pronounced, can be seen in the VF simulations of Bishop and Plank [36].) These results are consistent with the following experimental findings: (i) PS clustering related to trabeculae in swine [41] and (ii) filament anchoring to thinnest regions in sheep atria [52].

**Figure 9:**
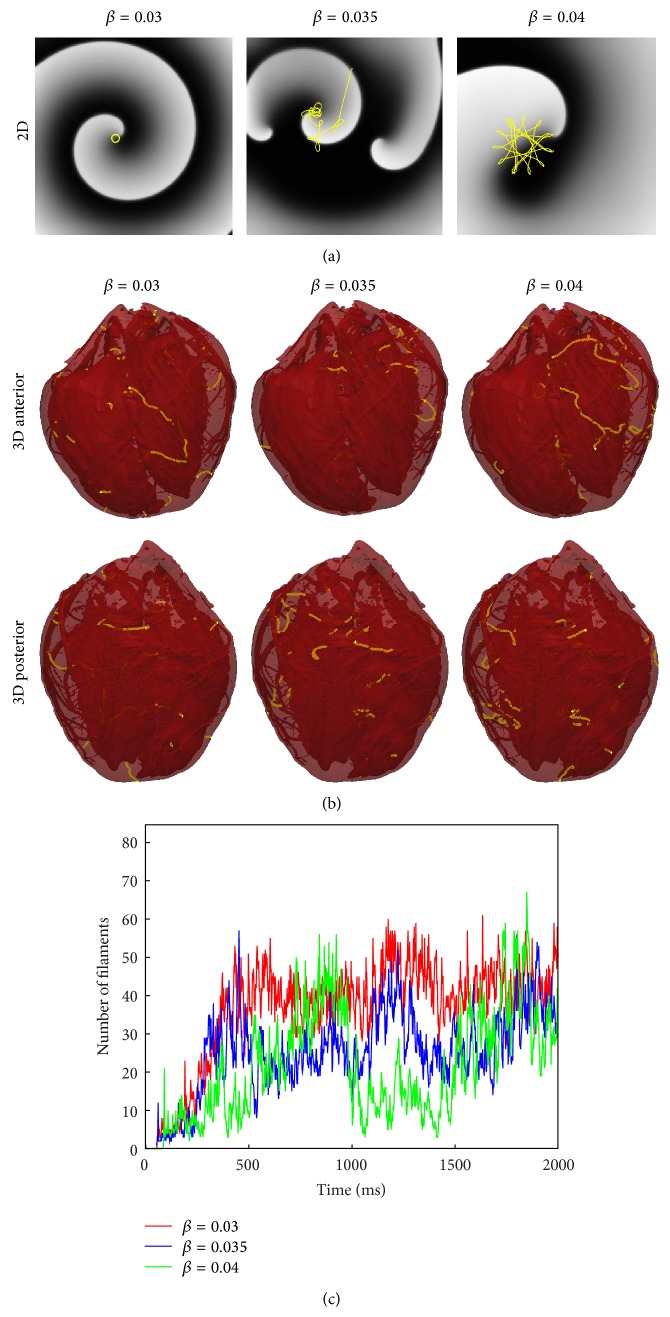
Relationship between 2D spiral wave dynamics and VF. (a) Results from 2D simulations (voltage snapshots in grayscale, tip trajectories in yellow). A rigidly rotating spiral wave (circular tip trajectory) is observed for *β* = 0.03; a meandering tip for *β* = 0.04, however for *β* = 0.035 break-up occurs. (b) Example of snapshots of filaments from corresponding 3D simulations (*t* = 1800 ms). Break-up occurs for all three parameter choices. (c) Number of filaments as a function of time. (Note: the relationship between number of filaments and number of rotors is discussed in Section 5.)

Finally, to investigate the role of our choice of ionic model on the observed filament dynamics presented here, we varied model parameters in the neighbourhood of the original parameter set, as shown in Figure 9. As mentioned in Section 2, the simulation of VF was performed with the parameter *β* in (1), which controls APD, taking the value 0.035/mV. This value was chosen because it exhibited spiral wave break-up in 2D simulations (Figure 9). To investigate the role of 2D instability (i.e., spiral wave break-up) on fibrillatory dynamics in the whole heart, we chose alternative parameter values that did not give rise to break-up but instead exhibited rigidly rotating (*β* = 0.03) and meandering (*β* = 0.04) spiral waves. However, in 3D whole heart simulations, spiral wave break-up (i.e., VF) occurred with* all three* parameter choices. Surprisingly, the complexity of simulated VF was similar for all three parameter choices, as quantified by counting the number of distinct filaments as a function of time. These results will be discussed in detail in Section 5.

## 5. Discussion

Much remains unknown about intramural electrical activity during ventricular fibrillation. In this paper, we have shown for the first time examples of filament dynamics during simulated VF in a realistic biventricular heart geometry with fine anatomical detail, such as vessels and trabeculae. One of the reasons for the lack of such examples in the literature is the technical difficulty in computing, analysing, visualising, and presenting such data. Overall, and as shown in Figure 9, we observed a large number of filaments (~30) during simulated VF. The filaments were highly dynamic in that their shapes and numbers rapidly varied. As shown in Figures 4 and 5, filaments tended to be fairly long and highly curved, leading to a variety of complex dynamics. This included the formation of closed filaments (i.e., scroll rings), residing completely within the heart walls. The length of filaments was often much greater than the thickness of the heart walls, as illustrated in Figure 3. Although we did observe filament rings pinch off from long filaments attached to the surface and rings merge with other filaments, we never observed linked rings. While similar filament dynamics have been observed in slabs, due to negative filament tension [53, 54] and twistons [25], it is not straightforward and perhaps impossible to translate results from 3D slab to the whole heart, especially with complex anatomical structures.

Although the number of filaments in our simulations is higher than reported by Bishop and Plank [36], there are several factors to be considered in the interpretation of our results. First and foremost, it should be noted that, as in experiments [21], the reentrant sources (filaments here, surface phase singularities in [21]) were mostly short-lived. Gray et al. [21] found that only about 20% of singularities lasted more than one rotation and hence formed rotors that actually sustain VF. We cautiously estimate the total number of rotors (i.e., sources) maintaining the simulated VF as approximately 4–8 (after 1 second), although the *β* = 0.04 simulation did exhibit a transient period (from 1 to 1.5 seconds) in which only ~10 filaments were present. We doubt that the majority of the short-lived filaments in our simulations (resulting from either intrinsic dynamics or anatomical heterogeneities) play a role in VF* maintenance*; however, it is extraordinarily difficult to discern the “symptoms” (filaments that do not form rotors) from the “cause” (rotors). Second, filaments necessarily break into “separate” filaments as they pass “through” vessels, increasing filament count. Supporting this notion is the fact that Bishop and Plank [36] found an approximately 40% decrease in the number of filaments as a result of removing the fine structure, with no change in the number of surface PSs (using the same heart mesh used here). Third, the APD in our model is unphysiologically short, which directly affects the wavelength and possibly the number of filaments. However, we believe that CV in our (and others') simulations of VF may be significantly greater than what is observed in experiments [46], so that the differences in wavelength in Figure 1 are smaller than expected.

The spatiotemporal patterns on the heart surface during our simulated VF qualitatively resemble those recorded from the surface of isolated rabbit hearts as shown in Figure 1, except for a difference in cycle length arising from the unphysiologically short APD in our model. Reducing APD was necessary because our model, like others, does not adequately represent the actual rate adaptation of wavelength that occurs during the transition to VF, although it is also possible that wave dynamics during VF are fundamentally different compared to during pacing or VT [55]. The VF cycle length in our simulations is approximately 35 ms versus approximately 100 ms in optical mapping recordings; however, it is important to note that the majority of high-resolution experimental recordings of VF (including those in Figure 1) have been carried out using uncoupling agents that affect action potential characteristics and arrhythmia dynamics [50]. In fact, the VF cycle length in isolated rabbit hearts is approximately 60–80 ms [51], significantly shorter than that observed in optical mapping experiments with uncoupling agents. We have performed an additional simulation of VF for which the cycle length matches optical mapping experiments but inactivation of the *I*_Na_ model was altered in order to obtain VF (results not presented), and the total number and dynamics of the resulting filaments during VF was found to be qualitatively similar to those presented here, suggesting that cycle length does not strongly impact the inferences drawn in this paper.

Rigorous validation and verification of cardiac electrophysiological simulations and VF, in particular, is extraordinarily difficult [56]. For example, in addition to the fact that uncoupling agents influence electrophysiology, optical mapping systems record fluorescence (not transmembrane potential), which has been shown to be significantly affected by intramural light scattering (although this effect can be included into simulations [57]). Somewhat akin to Heisenberg's uncertainty principle, it is prohibitively difficult to make high-resolution measurements during VF without disturbing the natural behaviour. As another example, Valderrábano et al. [41] implicated anatomical structure as anchoring points for filaments, but the majority of the data that this conclusion is based on are from recordings from the cut edges of transmural wedges in which the surface layer closest to the camera is severely disrupted. Verification (as defined in Section 2) of whole heart VF simulations introduces further challenges. While the computational mesh used is very high resolution (125 *μ*m) and is much finer than those used in most other cardiac modelling studies (typically 200–400 *μ*m), the mesh resolution is still in the range at which discretisation errors influence simulation results [44, 58, 59]. Simulation (and postprocessing) is currently infeasible using a finer mesh of the same geometry, and it remains to be proven that the discretisation is not affecting the observed filament dynamics.

Interestingly, simulations for model parameters which gave rise to stable and meandering spiral waves in 2D resulted in VF in the whole heart. The specific mechanisms of filament dynamics in our simulations is difficult to ascertain. The fact that we observed many filaments which grew and many filaments that were longer than the wall thickness suggests that negative tension may be involved. However, another possible cause of break-up is heterogeneous conductivity including rotational anisotropy, which may give rise to twistons. Here we discuss these possible mechanisms individually.

To determine the sign of filament tension, we performed scroll ring simulations in a cylindrical geometry, utilising rotational symmetry and a cylindrical coordinate system, and with isotropic conductivities. We found that* positive* filament tension (scroll wave shrinking) resulted from *β* = 0.03 and 0.04 (results not presented). It was not possible to determine the tension for the case *β* = 0.035 due to spiral wave break-up. Thus, although we cannot rule out the role of negative filament tension for *β* = 0.035, we demonstrate that filament tension is not the primary cause of scroll wave break-up in 3D in our simulations. Therefore, our results suggest that either the biventricular geometry and/or heterogeneous conductivity, including both those as a result of internal structures and rotational anisotropy, act to destabilise reentrant waves and sustain VF. An important previous result that we believe warrants further investigation was carried out by Alonso et al. [53] who showed that heterogeneity in cell-to-cell coupling can cause break-up in 3D in models exhibiting positive filament tension.

**Figure 10:**
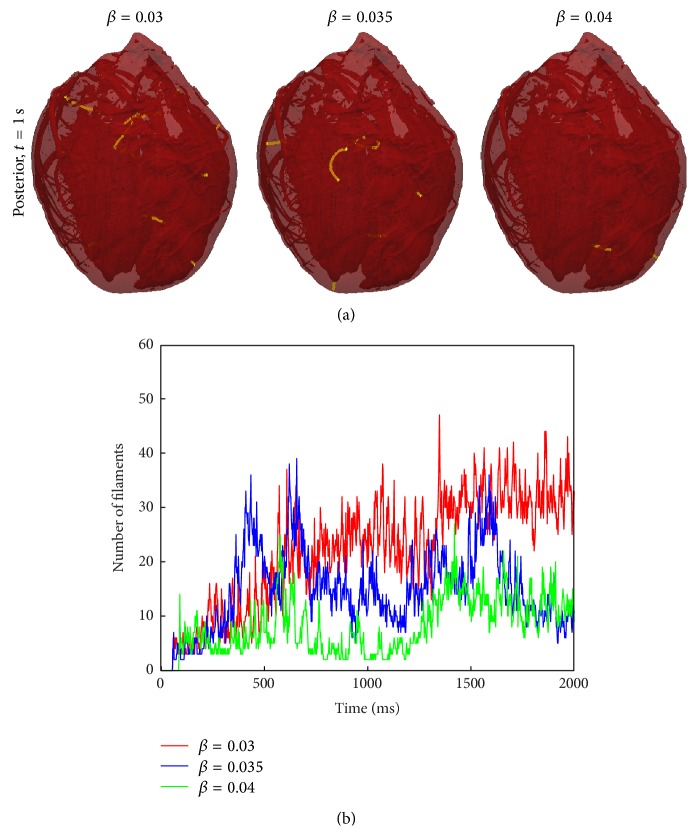
Number of filaments and snapshots at *t* = 1000 ms, using* isotropic* conductivities.

To study the role of rotational anisotropy, we repeated the whole heart simulations using an* isotropic* conductivity tensor. The best choice of conductivity value is not a straightforward decision. We chose a value of *σ* = *χ𝒞*_*m*_ *D* in all directions, where $D=\sqrt{D_{L}D_{T}}$, which corresponds to a local scaling of the microstructural coordinate system that preserves surface area in any plane parallel to the fibre direction. Effectively, the heart surface area is conserved, but transmural thickness is decreased. As partial justification for this choice, we note that the surface transmembrane voltage profiles just before the S2 stimulus were similar for the isotropic compared to anisotropic simulations. The results from the anisotropic (Figure 9) and isotropic simulations (Figure 10) illustrate similar qualitative behaviour in regard to the number of filaments and VF wave dynamics. However, the average number of filaments between *t* = 1 s and *t* = 2 s was decreased by 32% (*β* = 0.03), 50% (*β* = 0.035), and 55% (*β* = 0.04) in the isotropic simulations. These results suggest that twistons alone are not the primary destabilising factor during VF.

Overall, our study suggests that VF dynamics in the whole heart can be extremely complex and needs to be understood in relation to cell dynamics* and* heart structure. The biventricular anatomy alone gives rise to epicardial breakthrough patterns over the septum and many “hidden” filaments in the septum (as illustrated), as well as highly curved fibres at the septum wall junctions. The fact that VF occurred for parameter sets *β* = 0.03 and 0.04 implies that the heart structure (biventricular geometry and/or heterogeneous conductivity) acts to destabilise reentrant waves and thus may play a large role in VF dynamics. Surprisingly, the filament dynamics were similar for parameter sets that did and did not exhibit break-up in 2D. Our results also suggest a role of fine-scale structure in VF, which requires further investigation. We observed filament anchoring to the thin RV apex, although we did not observe any other direct evidence of anchoring. We also found a high density of filaments in the valleys between endocardial trabeculae (Figure 8) and evidence of vessels affecting filament dynamics (Figure 7 and supplementary movie).

In conclusion, we want to emphasise that both functional instabilities (resulting from cell dynamics) and anatomical structure may play important roles in the initiation and maintenance of VF. For example, the choice of the specific cell model will determine not only the period and wavelength of reentrant waves, but also the sign of filament tension and whether spiral wave break-up occurs in uniform 2D sheets. While Bishop and Plank [36] concluded that fine structure does not play a role in VF dynamics, it should be noted that they came to this conclusion by correlating filament locations during arrhythmias with geometrical structures and they did not study the* mechanism(s)* of break-up in the two ionic models they studied. It is unknown whether these models exhibited spiral wave break-up in 2D or negative filament tension. Our results suggest that further study of how fine-scale structure specifically affects wave propagation in the heart is warranted, including the assessment of mesh convergence. We believe that the relationship of the size of anatomical structures to such functional quantities as wavefront width, spiral wave core size, critical curvature for propagation, and liminal length holds the keys to understanding VF dynamics in the whole heart.

## Supplementary Material

The first movie (apex_RV_filament.mp4) focuses on the apical portion of the heart (anterior view). The mesh is translucent so that the endocardial surface and transmural vessels can be seen. The movie shows filament behaviour in this region from t=200ms to t=500ms. A small stable filament at the bottom of the right ventricle can be seen, and compared to more dynamic filaments in the left ventricle. This movie corresponds to Figure 6 in the paper
The second movie (filaments_and_vessel.mp4) corresponds to Figure 7 and shows possible interaction between a filament and coronary vessels in the left ventricular wall.
